# Seminal vesicle abnormalities following prostatic artery embolization for the treatment of benign prostatic hyperplasia

**DOI:** 10.1186/s12894-018-0407-7

**Published:** 2018-10-24

**Authors:** Jin Long Zhang, Kai Yuan, Mao Qiang Wang, Jie Yu Yan, Yan Wang, Guo Dong Zhang

**Affiliations:** 1 School of Medicine, Nan Kai University, 94 Wei-jin Rd, Tianjin, 300071 People’s Republic of China; 2 Department of Interventional Radiology, Chinese PLA General Hospital, 28 Fu-xing Rd, Beijing, 100853 People’s Republic of China

**Keywords:** Angiography, Benign prostatic hyperplasia, Prostate artery embolization, Seminal vesicle haemorrhage, Seminal vesicle ischaemia

## Abstract

**Background:**

Prostatic artery embolization (PAE) has been proved effective in the treatment of lower urinary tracts (LUTS) secondary to benign prostatic hyperplasia (BPH) with low complications, and most of the them are due to non-target embolization of adjacent organs, such as bladder, rectum, seminal vesicles and penis. Aim of this study was to present seminal vesicle (SV) abnormalities following prostatic artery embolization (PAE) for the treatment of symptomatic benign prostatic hyperplasia.

**Methods:**

We reviewed 139 BPH patients who received PAE during the period of February 2009 and January 2015 at a single institution, highlighting seminal vesicle abnormalities and their clinical relevance after PAE. PAE was performed using 90~ 180-μm (mean 100-μm) polyvinyl alcohol foam particles.

**Results:**

Nine of 139 patients with SV abnormalities (6.5%) were identified by magnetic resonance imaging (MRI), including subacute haemorrhage in 3 patients and ischaemia in 6 patients. Using cone-beam computed tomography (CB-CT), the seminal vesicle arteries were identified 8 of the 9 patients. All 9 patients complained of a few episodes of mild haematospermia during the 1–4 weeks after PAE; the haematospermia disappeared spontaneously without any treatment.

**Conclusion:**

SV haemorrhage and ischaemia may occur after PAE, and these patients may present with transient and self-limited haematospermia.

## Background

Prostatic artery embolization (PAE) has been adopted as a minimally invasive therapeutic modality for the treatment of lower urinary tract symptoms (LUTS) following benign prostatic hyperplasia (BPH) [1–10]. However, a recent systematic review suggest that PAE is inferior to standard treatment methods, such as open prostatectomy (OP) or transurethral resection of the prostate (TURP), and PAE is still considered an experimental treatment modality [11]. Complications of PAE are low and are primarily related to non-target embolization of other arteries, such as the vesical, rectal, and dorsal arteries of the penis. Major complications have been rare, with only two cases of bladder focal necrosis [1, 7]. Minor adverse events after PAE have occurred in, cumulatively, 11% of patients [1, 2, 7–10] and have included urinary tract infections, transient haematuria, transient haematospermia, a small amount of rectal bleeding, ischaemic rectitis, and balanitis.

Haematospermia, an uncommon clinical event, has occurred in 5.9–16% of cases after PAE [1, 3, 5, 10]. We hypothesize that haematospermia secondary to PAE may be associated with seminal vesicle (SV) ischaemia and haemorrhage, resulting from non-target embolization. Herein, we report nine cases of SV abnormalities after PAE, including SV ischaemia in 6 patients and SV haemorrhage in 3 patients, identified by magnetic resonance imaging (MRI) follow-up.

## Methods

### Patients

Between February 2009 and January 2015 in our institution, a total of 139 patients (mean age, 72.0 years±10.5 [standard deviation]) diagnosed with moderate or severe LUTS (International Prostate Symptoms Score [IPSS] > 18 points, quality of life [QoL] score > 3, and/or urinary retention with urinary catheter removal failure) due to BPH who were refractory to medical treatment for at least 6 months underwent PAE.

### PAE protocols

The selection criteria included patients with a diagnosis of severe LUTS, negative screening for prostate cancer, prostate volume (PV) > 40 mL measured by MRI, and bladder outlet obstruction (BOO) confirmed by urodynamic examination, peak urinary flow rate (Qmax) < 12 mL/sec, and PVR post-void residual urine (PVR) > 150 mL evaluated by ultrasound, biopsy was performed to rule out prostate malignant if PSA level > 4.0 ng/mL. The patient selection was evaluated by a multidisciplinary team that included urologists, anaesthesiologists, and interventional radiologists. Exclusion criteria included pelvic malignancy, chronic renal failure, large bladder diverticula (> 5 cm), active urinary tract infection, large bladder stones (> 2 cm), unregulated coagulation parameters, neurogenic bladder, allergy to intravenous contrast media, detrusor failure and urethral stricture diagnosed through pressure flow studies or urethrography [1, 9].

The preparative clinical observation included IPSS, QoL, peak urinary flow rate (Qmax), post-void residual volume (PVR), international index of erectile function short form (IIEF-5) score, and PV before PAE and at 1, 3, 6 and every 6 months after the procedure. All patients underwent 1.5-T multiparametric enhanced MRI (GE Healthcare, Milwaukee, Wisconsin, USA) of the prostate to measure PV and to rule out cancer before PAE using a phased-array 12-channel body coil. For each patient, the MRI protocol was the same, including axial, coronal, and sagittal T2-weighted imaging (T2WI) and contrast- and non-contrast enhanced T1-weighted imaging (T1WI).

### Embolization technique

The details of the procedure of PAE have been described previously [10]. The PAEs were performed by two senior interventional radiologists (M.Q.W. and K. Y., with 26 and 12 years of vascular and interventional radiology experience, respectively), using a therapeutic angiography unit equipped with a digital flat-panel detector system (INNOVA 4100 IQ; GE Healthcare, Milwaukee, Wisconsin, USA). PAE was performed under local anaesthesia through a single right femoral approach using a 4-Fr vascular sheath (Radifocus, Terumo, Japan). Digital subtraction angiography (DSA) and cone-beam computed tomography (CB-CT) were performed to identify prostatic arteries (PAs). Embolization was performed with 100-μm non-spherical PVA particles (90~ 180-μm, PVA, Cook Incorporated, Bloomington, IN, USA). The endpoint of embolization was occlusion of the identifiable vessels supplying the prostate.

### Follow-up

Follow-up was performed at 1, 3, 6, and every 6 months after PAE by the interventionalists and the urologists. IPSS, QoL, IIEF-5, PSA, Qmax, PVR, and PV on MRI were evaluated at those dates to measure clinical and radiological changes after PAE.

### Imaging evaluation

All MR images were assessed independently by two radiologists (reader 1 and reader 2, with 11 years and 15 years of experience in interpreting body MR images, respectively) without knowing the outcomes of the PAE. If there was disagreement, the relevant MR images were reassessed by a third independent reader (reader 3, with 20 years of experience in interpreting body MR) to reach a consensus.

The procedural angiographic images, including DSA, rotational angiography, and CB-CT, were reviewed retrospectively by two interventional radiologists (G. D. Z. and M.Q.W., with 16 and 25 years of vascular and interventional radiology experience, respectively), highlighting the possibility of the blood supplying the SV (“vesiculo-deferential artery”). After independent interpretations were achieved, the differences in evaluations between the two radiologists were resolved by consensus.

## Results

### Peri-procedural outcomes

Nine cases of SV abnormalities (6.5%) after PAE, including SV ischaemia in 6 patients and SV haemorrhage in 3 patients, were identified by MRI follow-up. The baseline characteristics of the nine patients are provided in Table 1. PAE was performed bilaterally in the 9 patients, identifying a total of 13 prostatic arteries. Of these prostatic arteries, six were originated from the internal pudendal artery, and seven were originated from the gluteal-pudendal trunk. No immediately procedural complications occurred.

**Table 1:** Clinical Data Obtained before and at 12 Months after PAE (*N* = 9)

Patient	IPSS		QoL		PV(ml)		PSA(ng/ml)		Q_max_(ml/s)		PVR(ml)		IIEF-5	
	Pre	Post	Pre	Post	Pre	Post	Pre	Post	Pre	Post	Pre	Post	Pre	Post
1	28	6	6	1	79	39	4.6	3.4	7.0	15.0	70	0	16	18
2	26	5	5	0	72	44	3.9	3.0	8.5	16.0	50	0	19	19
3^a^	32	5	6	1	127	58	8.0	3.9	–	14.0	–	10	9	10
4	30	7	6	2	90	47	3.0	3.4	6.0	14.0	80	10	17	15
5	27	5	5	0	67	37	2.0	1.5	8.0	16.0	60	0	16	18
6	29	8	6	2	116	64	7.1	5.7	5.0	13.0	100	10	7	7
7	28	6	6	1	87	46	4.5	3.0	7.0	15.0	90	0	11	12
8	27	4	6	1	84	52	7.0	2.0	10.0	19.0	70	0	18	19
9	30	6	6	2	122	66	2.9	1.5	8.5	17.0	110	0	15	16
mean	28.6	6	5.8	1	93.8	50.3	4.8	3.1	7.5	15.4	74.3	3.8	14	15

^a^Patient with urinary retention before PA

### Imaging findings

**Fig. 1:**
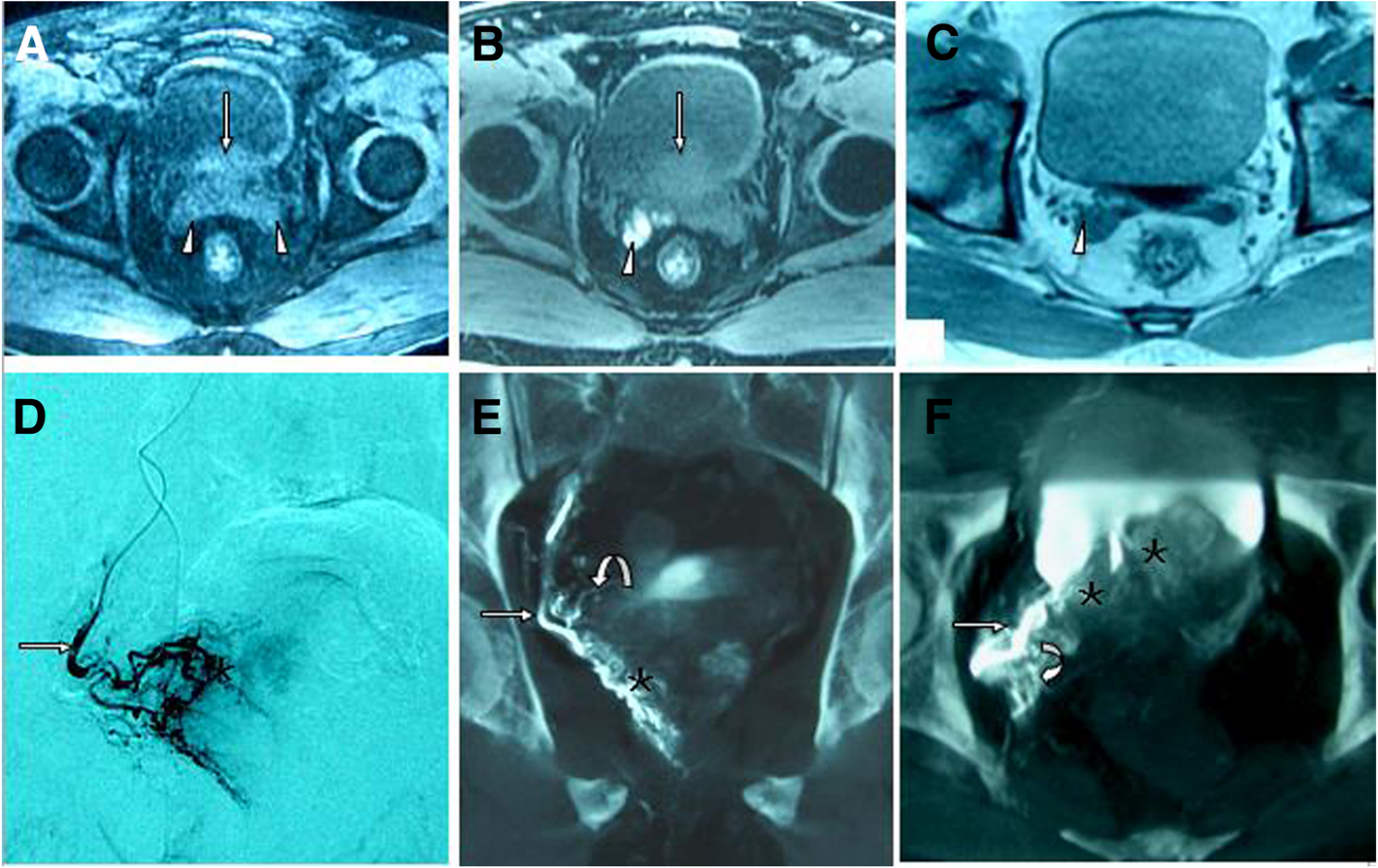
Seminal vesicle haemorrhage. Image from a 65-year-old man with lower urinary tract symptoms due to benign prostatic hyperplasia (BPH). He presented with mild haematospermia at 1 week after PAE that disappeared 4 weeks later without specific treatment. **a** Axial T1-weighted MR image obtained before PAE shows the normal appearance of the seminal vesicles (arrowheads) and BPH (straight arrow). **b** Axial T1-weighted MR image obtained 1 month after PAE shows high-intensity signals on the right side of the seminal vesicles (arrowhead), suggestive of haemorrhage, and BPH (straight arrows). **c** Axial T1-weighted MR image (without fat suppression) obtained 12 months after PAE shows iso-intensity signals on the right side of the seminal vesicles (arrowhead) and reduction in the size of the SVs. **d** Digital subtraction angiography (DSA) of the right prostatic artery (straight arrow) with same-side anterior oblique projection (35°) demonstrates contrast medium staining in the right prostate lobe (asterisk). **e** Cone-beam CT (CB-CT) with coronal view after catheterization of the right prostatic artery (straight arrow) demonstrates the small branches (curved arrow) supplying the seminal vesicles and contrast medium staining in the right prostate lobe (asterisk). **f** CB-CT with axial view after catheterization of the right prostatic artery (straight arrow) demonstrates the small branches (curved arrow) supplying the seminal vesicles (the seminal vesicle artery) and contrast medium staining in the right prostate lobe (asterisks)

Subacute SV haemorrhage was presented in 3 patients (Patient No. 1, 2, and 3). MRI at 1 month following PAE showed high-intensity signals on T1WI with low-intensity signals on T2WI in the SVs, suggesting typical subacute haemorrhage (Fig. 1a-b). These findings were not presented on the pre-procedural MRI. During the 3- to 12-month follow-up, these high-intensity signals on T1WI within the SVs became iso-intensity signals, with a reduction in the size of the SV, suggestive of SV atrophy (Fig. 1c). With retrospective analysis of the intra-procedural angiographic images, the CB-CT images showed that the small arteries branched proximally from the prostate arteries supplied to the SV (i.e., the seminal vesicle arteries) in 3 patients; however, these small arterial branches could not be identified on DSA (Fig. 1d-f).

**Fig. 2:**
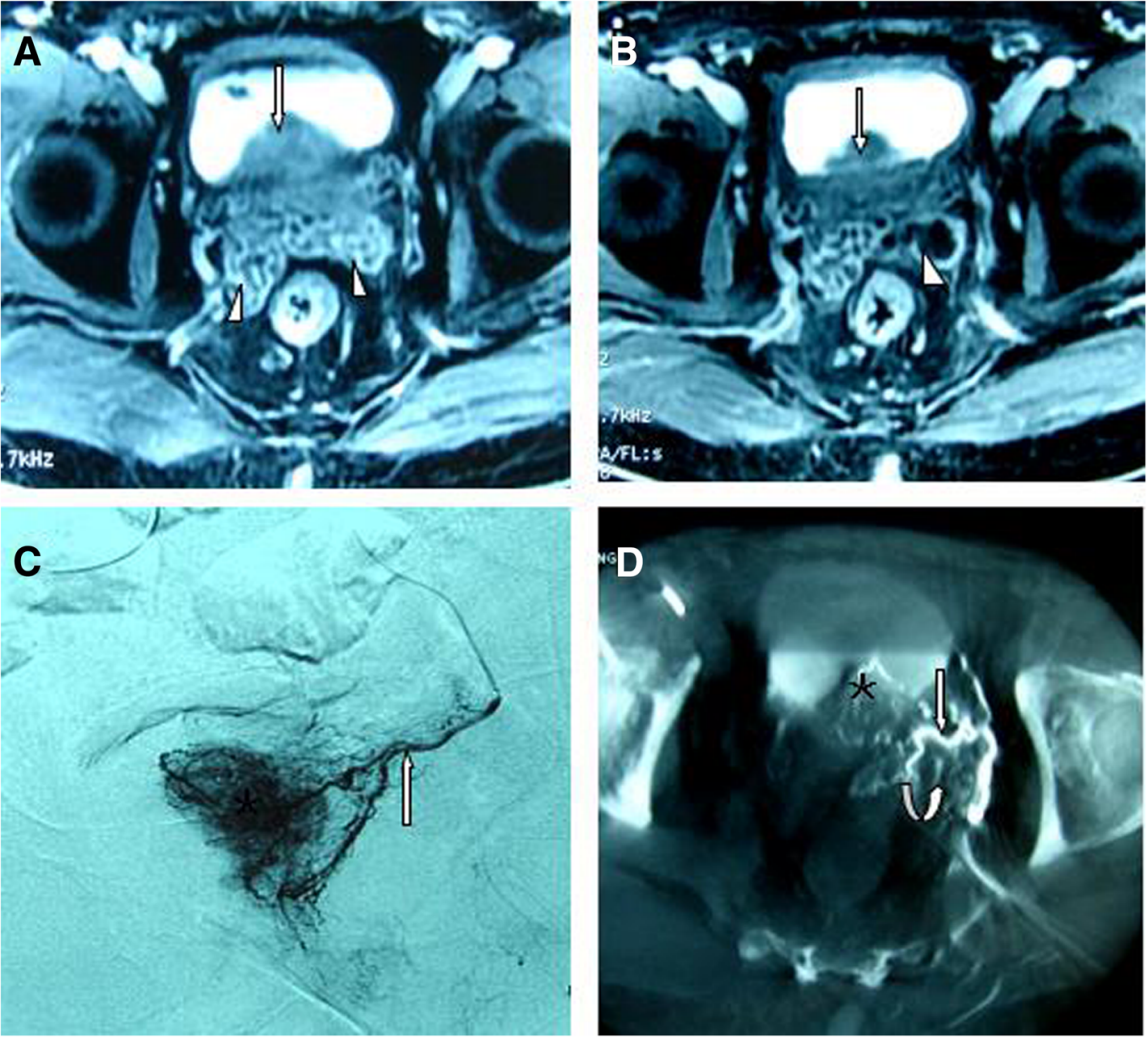
Seminal vesicle ischaemia. Image from a 72-year-old patient with lower urinary tract symptoms due to a large BPH (120 mL). **a** Axial contrast-enhanced T1-weighted MR image obtained before PAE shows a large benign prostatic hyperplasia (straight arrow) and normal seminal vesicles (arrowheads). **b** Axial contrast-enhanced T1-weighted MR image obtained 1 month after PAE shows reduction of the prostate (straight arrows) and hypoperfusion in the seminal vesicles (arrowhead), suggestive of ischaemia. **c** DSA of the left prostatic artery (straight arrow) with same-side anterior oblique projection (35°) demonstrates contrast-medium staining in the left prostate lobe (asterisk). **d** CB-CT with axial view after catheterization of the left prostatic artery (straight arrow) demonstrates the small branches (curved arrow) supplying the seminal vesicles (the seminal vesicle artery) and contrast medium staining in the left prostate lobe (asterisk)

**Fig. 3:**
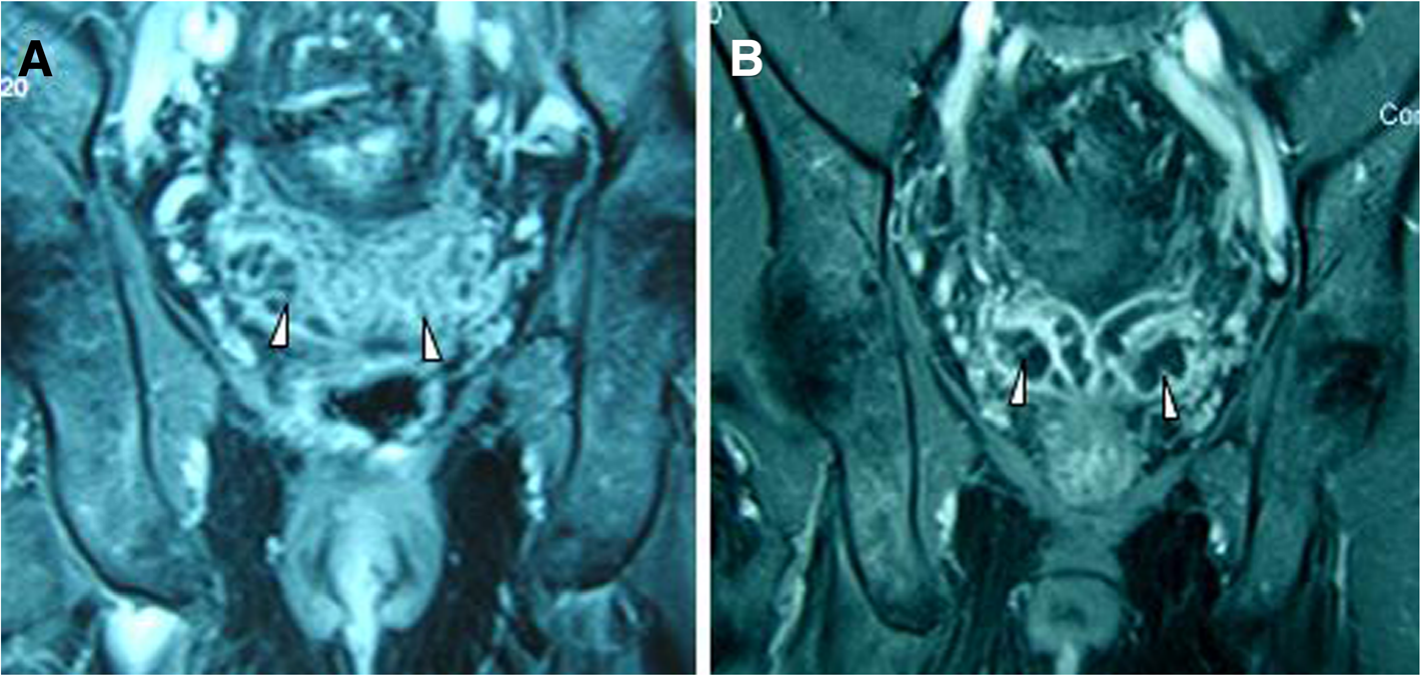
Seminal vesicle ischaemia. Image from a 69-year-old patient with lower urinary tract symptoms due to a large BPH (132 mL). **a** Coronal contrast-enhanced T1-weighted MR image obtained before PAE shows normal seminal vesicles (arrowheads). **b** Coronal contrast-enhanced T1-weighted MR image obtained at 1 month after PAE shows significant hypoperfusion in the seminal vesicles (arrowheads), suggestive of ischaemia

**Fig. 4:**
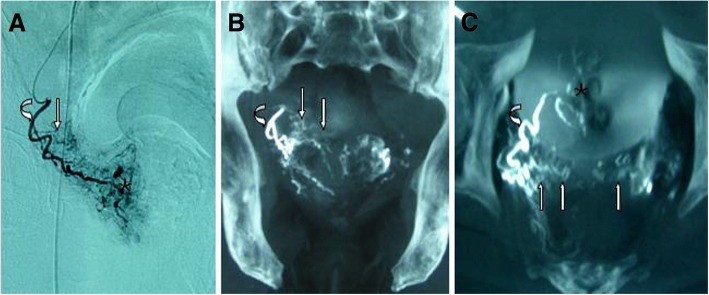
Images from the same patient as Fig. 3. **a** DSA of the right prostatic artery (curved arrow) with same-side anterior oblique projection (35°) demonstrates contrast-medium staining in the right prostate lobe (asterisk) and the small branches (straight arrow), which were suspected to be the seminal vesicle arteries. **b** CB-CT with coronal view after catheterization of the right prostatic artery (curved arrow) demonstrates the small branches (straight arrows) supplying the seminal vesicles. **c** CB-CT with axial view after catheterization of the right prostatic artery (curved arrow) demonstrates the small branches (straight arrows) supplying the seminal vesicles (the seminal vesicle arteries) and contrast medium staining in the prostate (asterisks)

SV ischaemia was presented in 6 patients (Patient Nos. 4–9). Contrast-enhanced T1WI images at 1 month following PAE showed obvious hypoperfusion in the seminal vesicles, suggestive of ischaemia (Figs. 2, 3 and 4). SV atrophy was also noted in those patients during the follow-up. The SV arteries could be identified in 5 of the 6 patients on the CB-CT but were difficult to identify on DSA.

### The relevant clinical findings

At the 1-month follow-up visit, all 9 patients complained a few episodes of mild haematospermia during 1–4 weeks after PAE; the haematospermia disappeared spontaneously without any treatment. Four of them had co-occurring mild macroscopic haematuria, but without evidence of bladder ischaemia on the MRI follow-up. Three cases had temporary anxiety due to the haematospermia. The remaining 130 patients had no complaints of haematospermia or haematuria; no abnormalities of the SVs were presented on the follow-up MRI.

The mean follow-up time of the 9 patients was 22 months (range, 14–36 months). The mean IPSS (pre-PAE vs post-PAE 28.6 vs 6.0; *P* < 0.01), QoL (5.8 vs 1.0; *P* < 0.05), Qmax (7.5 vs 15.4; *P* < 0.01), PVR (74 mL vs 4 mL; *P* < 0.01), PV (94 mL vs 40 mL; *P* < 0.05), and PSA (4.8 ng/mL vs 3.1 ng/mL; *P* < 0.05) had significant differences compared with baseline, as shown in Table 1. The mean IIEF-5 had no significant difference from baseline (*P* = 0.8).

## Discussion

PAE is a safe and effective procedure with low morbidity in most cases [1, 7–13]. Although various complications of PAE, such as bladder ischaemia, urinary tract infections, balanitis, and ischaemic rectitis, have been reported, seminal vesicle abnormality after PAE is rare [1, 7, 14, 15]. In the present study, the incidence of SV abnormalities (haemorrhage and ischaemia) after PAE, identified by MRI follow-up, was 6.5%. Clinically, patients with SV haemorrhage or ischaemia usually present with haematospermia [16]. Pisco JM et al. [1] reported that transient haematospermia occurred in 7% of cases after PAE. De Assis AM et al. [5] reported that transient and self-limited haematospermia occurred in 5.9% of cases after PAE. Recently, Amouyal G et al. [12] evaluated 32 patients treated with PAE and reported that 3 patients (9%) experienced haematospermia during 3 days to 1 month after PAE. Bagla et al. [3] reported an incidence of 16% (3 of the 19 patients) self-limited haematospermia even using CB-CT.

Haematospermia secondary to PAE may be related to seminal vesicle ischaemia and haemorrhage resulting from non-target embolization. In the present cases, the SV abnormalities could be explained by embolic particle reflux, existence of a common trunk between the prostate capsule branch and the seminal vesicle artery, misidentification of the seminal vesicle artery as a capsular artery, or a prostato- seminal vesicle arterial anastomosis not detected angiographically at the time of embolization.

There is no specific therapeutic option in the treatment of seminal vesicle haemorrhage or ischaemia after PAE. However, bacterial inflammation may occur after bleeding in the seminal vesicles [16]. Therefore, antibiotic treatment is recommended once seminal vesicle haemorrhage or ischaemia has been confirmed by MRI. In addition, haematospermia after PAE may cause the psychological anxiety for the patient; therefore, it is necessary to explain the complication to patients before the procedure.

Knowledge of the detailed anatomy of the pelvic arteries is crucial for a safe and effective PAE and to avoid complications from non-targeted embolization of surrounding organs to yield better outcomes [1, 2, 17]. The blood supplies to the prostate, bladder, rectum, and penis have been reported previously in the literature [18–22]. However, little work appears to have been performed on the anatomy of the arterial circulation in the seminal vesicles; most of the standard textbooks even ignore the existence of a blood supply to this organ. In early studies in cadaveric specimens by Clegg EJ [23], he described that the seminal vesicle arteries were supplied by the “vesiculo-deferential artery”; the origin of this vessel is highly variable and includes the umbilical artery, internal pudendal artery, superior vesical artery, and prostato-vesical artery. Currently, there are no in vivo studies published in the literature documenting the imaging findings of SV artery anatomy.

In the present study, with retrospective reviews using DSA and CB-CT, we could identify the SV arteries, which presented with very small branches on the CB-CT images in 8 of the nine patients, which originated proximally from the prostatic artery. To prevent PAE-related SV complications, more studies are needed to understand the detailed anatomy of the SV arteries. From our experience, CB-CT performed intraoperatively using a three-dimensional arteriography with maximum-intensity projection is a useful tool for PAE procedures. Although the result is a low-quality image compared with that of conventional computed tomography, it provides good vessel identification when vessels cannot be visualized on arteriography [24].

## Conclusions

Although the SV abnormalities after PAE were not significant consequences, interventionalists should be aware of that non-targeted embolization of the seminal vesicles may be the cause of haematospermia. Long-term follow-up is needed to understand the long-term effects of seminal vesicle haemorrhage or ischaemia.

## Abbreviations

BPH: Benign prostatic hyperplasia
CB-CT: Cone-beam computed tomography
DSA: Digital subtraction angiography
IIEF-5: International Index of Erectile Function
IPSS: International Prostate Symptom Score
LUTS: Lower urinary tract symptoms
MRI: Magnetic resonance imaging
PAE: Prostatic artery embolization
PSA: Prostate-specific antigen
PV: Prostate volume
PVA: Polyvinyl alcohol particles
PVR: Post-void residual volume
Q_max_: Peak urinary flow rate
QoL: Quality of life

## References

[1] Pisco J, Campos Pinheiro L, Bilhim T, Duarte M, Rio Tinto H, Fernandes L, Vaz Santos V, Oliveira AG (2013). Prostatic arterial embolization for benign prostatic hyperplasia: short- and intermediate-term results. Radiology.

[2] Carnevale FC, da Motta-Leal-Filho JM, Antunes AA, Baroni RH, Marcelino AS, Cerri LM, Yoshinaga EM, Cerri GG, Srougi M (2013). Quality of life and clinical symptom improvement support prostatic artery embolization for patients with acute urinary retention caused by benign prostatic hyperplasia. J Vasc Interv Radiol.

[3] Bagla S, Martin CP, van Breda A, Sheridan MJ, Sterling KM, Papadouris D, Rholl KS, Smirniotopoulos JB, van Breda A (2014). Early results from a United States trial of prostatic artery embolization in the treatment of benign prostatic hyperplasia. J Vasc Interv Radiol.

[4] Russo GI, Kurbatov D, Sansalone S, Lepetukhin A, Dubsky S, Sitkin I, Salamone C, Fiorino L, Rozhivanov R, Cimino S, Morgia G (2015). Prostatic arterial embolization vs open prostatectomy: a 1-year matched-pair analysis of functional outcomes and morbidities. Urology.

[5] de Assis AM, Moreira AM, de Paula Rodrigues VC, Yoshinaga EM, Antunes AA, Harward SH, Srougi M, Carnevale FC (2015). Prostatic artery embolization for treatment of benign prostatic hyperplasia in patients with prostates > 90 g: a prospective single-center study. J Vasc Interv Radiol.

[6] Sun F, Crisóstomo V, Báez-Díaz C, Sánchez FM (2016). Prostatic artery embolization (PAE) for symptomatic benign prostatic hyperplasia (BPH): part 1, pathological background and clinical implications. Cardiovasc Intervent Radiol.

[7] Lebdai S, Delongchamps NB, Sapoval M, Robert G, Amouyal G, Thiounn N, Karsenty G, Ruffion A, de La Taille A, Descazeaud A, Mathieu R (2016). Early results and complications of prostatic arterial embolization for benign prostatic hyperplasia. World J Urol.

[8] Carnevale FC, Iscaife A, Yoshinaga EM, Moreira AM, Antunes AA, Srougi M (2016). Transurethral resection of the prostate (TURP) versus original and PErFecTED prostate artery embolization (PAE) due to benign prostatic hyperplasia (BPH): preliminary results of a single center, prospective, urodynamic-controlled analysis. Cardiovasc Intervent Radiol.

[9] Wang MQ, Wang Y, Yan JY, Yuan K, Zhang GD, Duan F, Li K (2016). Prostatic artery embolization for the treatment of symptomatic benign prostatic hyperplasia in men ≥75 years: a prospective single-center study. World J Urol.

[10] Wang MQ, Guo LP, Duan F, Yuan K, Zhang GD, Li K, Yan JY, Wang Y, Kang HY (2016). Prostatic arterial embolization for the treatment of lower urinary tract symptoms caused by benign prostatic hyperplasia: a comparative study of medium- and large-volume prostates. BJU Int.

[11] Shim SR, Kanhai KJ, Ko YM, Kim JH (2017). Efficacy and safety of prostatic arterial embolization: systematic review with meta-analysis and meta-regression. J Urol.

[12] Amouyal G, Thiounn N, Pellerin O, Yen-Ting L, Del Giudice C, Dean C, Pereira H, Chatellier G, Sapoval M (2016). Clinical results after prostatic artery embolization using the PErFecTED technique: a single-center study. Cardiovasc Intervent Radiol.

[13] McWilliams JP, Kuo MD, Rose SC, Bagla S, Caplin DM, Cohen EI, Faintuch S, Spies JB, Saad WE, Nikolic B (2014). Society of InterventionalRadiology. Society of interventional radiology position statement: prostate artery embolization for treatment of benign disease of the prostate. J Vasc Interv Radiol.

[14] Moreira AM, Marques CF, Antunes AA, Nahas CS, Nahas SC, de Gregorio Ariza MA, Carnevale FC (2013). Transient ischemic rectitis as a potential complication after prostatic artery embolization: case report and review of the literature. Cardiovasc Intervent Radiol.

[15] Pisco JM, Rio Tinto H, Campos Pinheiro L, Bilhim T, Duarte M, Fernandes L, Pereira J, Oliveira AG (2013). Embolisation of prostatic arteries as treatment of moderate to severe lower urinary symptoms (LUTS) secondary to benign hyperplasia: results of short- and mid-term follow-up. Eur Radiol.

[16] Furuya S, Furuya R, Masumori N, Tsukamoto T, Nagaoka M (2008). Magnetic resonance imaging is accurate to detect bleeding in the seminal vesicles in patients with hemospermia. Urology.

[17] Carnevale FC, Antunes AA (2013). Prostatic artery embolization for enlarged prostates due to benign prostatic hyperplasia. How I do it. Cardiovasc Intervent Radiol.

[18] Garcia-Monaco R, Garategui L, Kizilevsky N, Peralta O, Rodriguez P, Palacios-Jaraquemada J (2014). Human cadaveric specimen study of the prostatic arterial anatomy: implications for arterial embolization. J Vasc Interv Radiol.

[19] Bilhim T, Pisco JM, Rio Tinto H, Fernandes L, Pinheiro LC, Furtado A, Casal D, Duarte M, Pereira J, Oliveira AG, O'Neill JE (2012). Prostatic arterial supply: anatomic and imaging findings relevant for selective arterial embolization. J Vasc Interv Radiol.

[20] de Assis AM, Moreira AM, de Paula Rodrigues VC, Harward SH, Antunes AA, Srougi M, Carnevale FC (2015). Pelvic arterial anatomy relevant to prostatic artery embolisation and proposal for angiographic classification. Cardiovasc Intervent Radiol.

[21] Bilhim T, Tinto HR, Fernandes L, Martins Pisco J (2012). Radiological anatomy of prostatic arteries. Tech Vasc Interv Radiol.

[22] Carnevale FC, Soares GR, de Assis AM, Moreira AM, Harward SH, Cerri GG (2017). Anatomical variants in prostate artery embolization: a pictorial essay. Cardiovasc Intervent Radiol.

[23] CLEGG EJ (1955). The arterial supply of the human prostate and seminal vesicles. J Anat.

[24] Bagla S, Rholl KS, Sterling KM, van Breda A, Papadouris D, Cooper JM, van Breda A (2013). Utility of cone-beam CT imaging in prostatic artery embolization. J Vasc Interv Radiol.

